# Case Report: Infant-onset Degos disease with nervous system involvement and a literature review

**DOI:** 10.3389/fped.2024.1374150

**Published:** 2024-07-05

**Authors:** Xin-Wei Shi, Jiang-Hong Deng, Cai-Feng Li

**Affiliations:** Department of Rheumatology, National Center for Children’s Health, Beijing Children’s Hospital, Capital Medical University, Beijing, China

**Keywords:** Degos disease, malignant atrophic papulosis, infantile onset, neurological involvement, prognosis

## Abstract

Degos disease also known as malignant atrophic papulosis (MAP), is an autoinflammatory disease that mainly affects small- to medium-sized arteries. Gastrointestinal and nervous system are most commonly affected systems. Herein, we reported a case of Degos disease with disease onset during infantile and had severe neurological involvement.

## Introduction

Degos disease, also known as malignant atrophic papulosis (MAP), is an autoinflammatory disease that mainly affects small- to medium-sized arteries (1). Initially described in 1941, it was later reclassified as malignant atrophic papulosis due to its involvement in multiple organs. While Degos disease commonly manifests in the fourth decade of adulthood, there have been reported cases in infants (2). The first infantile Degos disease was reported by Henkind and Clark, who had disease onset at three weeks of life and died due to neurological complications. Subsequent cases with neurological involvement have been reported, alluding to an overall unfavorable prognosis. Until now, more than 200 cases of Degos disease have been reported, and approximately 30 cases of childhood Degos disease have been published. Despite its rarity, there are no reported cases of infantile Degos disease in China. This report details a patient exhibiting severe neurological manifestations with an overall grim prognosis.

## Case presentation

**Figure 1:**
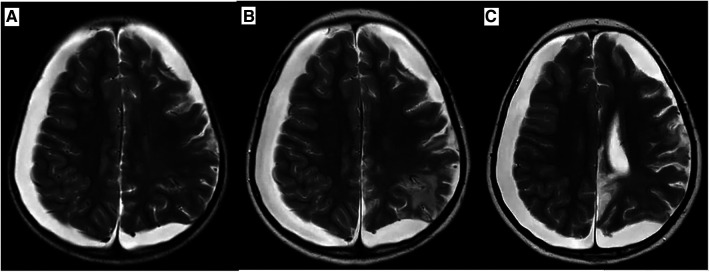
Axial T2 weighted Brain MR image during disease progression. (**A**) Brain MRI at first visit revealed atrophy of left hemisphere, subdural effusion, calcification foci, hemorrhagic region, ischemic region and vasculitis. (**B**) Brain MRI after one month of treatment which revealed the size of ischemic region increased. (**C**) Brain MRI after one and a half months of treatment which revealed medium amount of subdural effusion and size of ischemic region increased.

**Figure 2:**
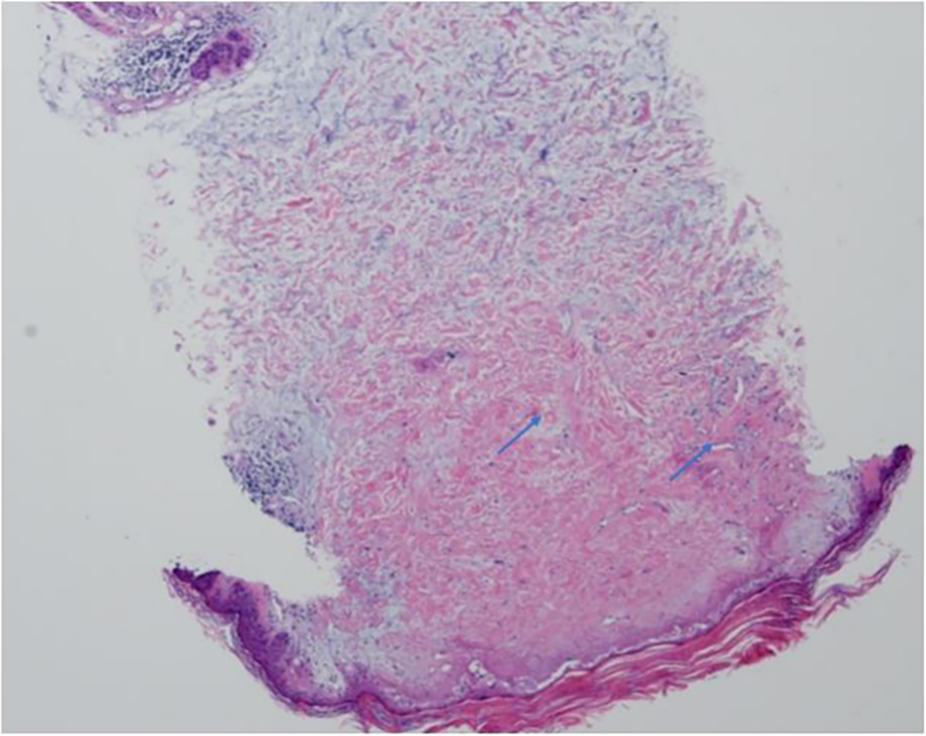
Histopathological examination of skin lesions showed dermis necrosis , thickness of vascular wall and perivascular lymphocytic infiltration (arrow) (H&E,20X).

**Figure 3:**
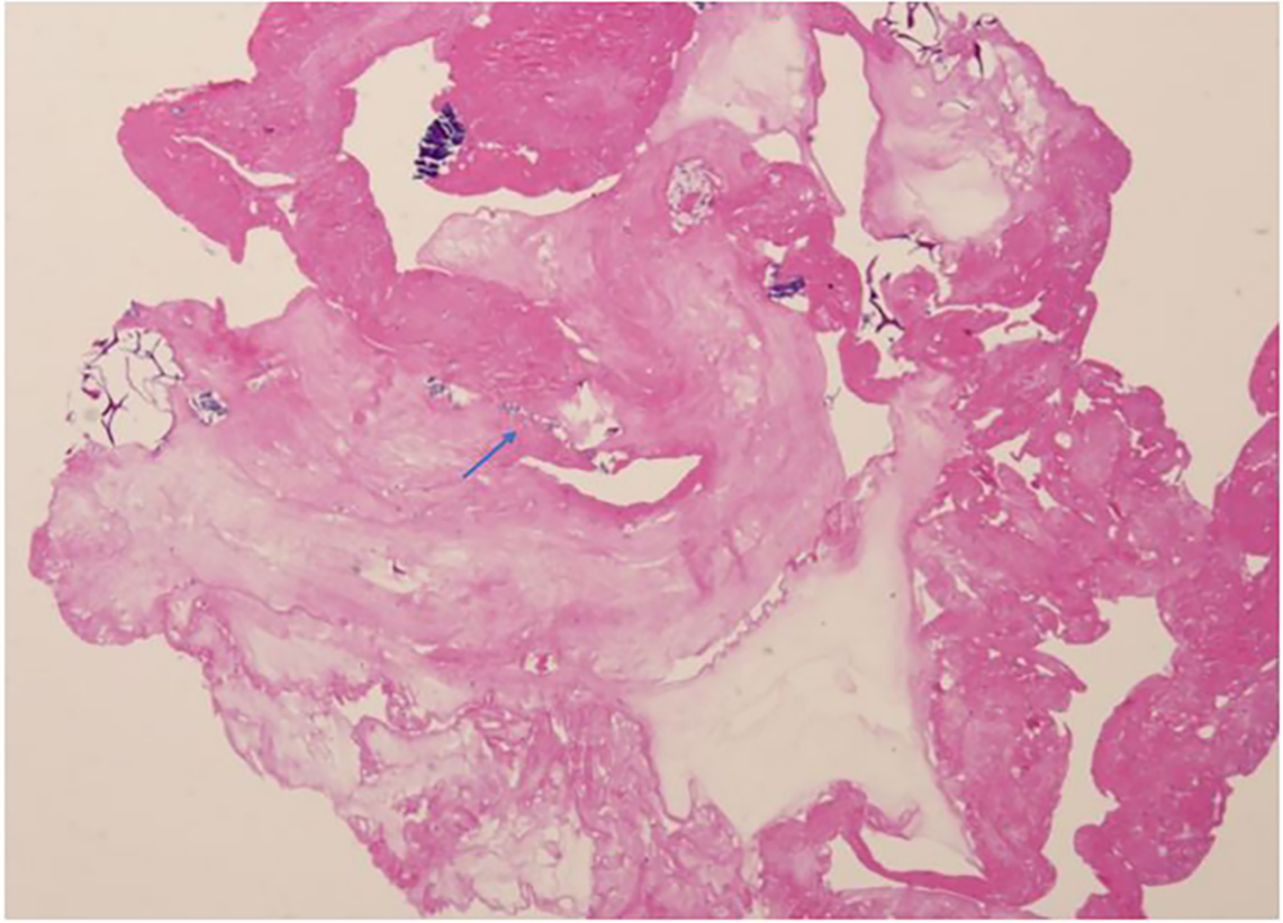
Histopathological examination of subdural effusion inflammation cells infiltration (arrow) (H&E,20X).

A 6-year-old Chinese girl presented with three months of numbness and two months of weakness in her extremities. Three months before admission, the patient developed right foot numbness and subsequently recovered. Two months before admission, weakness in the right lower limb developed, which manifested as claudication. Later, her symptoms worsened to flaccid paralysis of the bilateral limbs, and the patient was referred to the neurological department of the local hospital. Physical examination revealed right peripheral facial paralysis, right eye ptosis and flaccid paralysis of bilateral limbs. Laboratory examinations, such as blood cell levels, were unremarkable, and the patient was presented with normal liver and kidney function. The erythrocyte sedimentation rate (ESR) was 33 mm/h. Cerebrospinal fluid (CSF) protein concentration was 311 mg/dl. CSF revealed the presence of pleocytosis (25 × 10^6^/l, normal range 0–15 ×10^6^/l), with lymphocyte predominance. CSF IgM was 0.638 mg/ml. Cultivation of CSF and autoimmune encephalitis-associated antibodies was negative. Brain Magnetic Resonance Imaging (MRI) showed subdural effusion. The patient received two doses of methylprednisolone pulse and intravenous immunoglobulin (IVIG) therapy, but the disease progressed to incontinence of the stool and urine and involuntary movement of both lower extremities. The patient was subsequently transferred to the neurological department of our hospital to determine the etiology of the disease. On examination, right facial paralysis was noted. Decreased muscle strength and involuntary movement of both lower limbs were observed. The sensation of both lower limbs was lost to pin, touch, temperature and position. Cerebellar signs and cranial nerve examination were negative. Deep tendon reflexes showed hyporeflexia. Skin examination revealed dark-red papules throughout the body with a white atrophic center. After a detailed inquiry of her medical history, the patient had round to oval dark-red papules with white calcification centers on her left forefinger and dorsal wrist at the age of 20 days. These papules progressed throughout the body, subsided within two weeks and subsequently recurred. Additional laboratory results were as follows: routine blood and urine tests were unremarkable, and the patients presented with normal liver and kidney function. Repeated ESR was 10 mm/h. Cerebrospinal fluid (CSF) protein concentration was 2,215 mg/dl which is higher than before. CSF revealed the normal range of granulocyte. Her plasma D-dimer level was 0.76 mg/l (normal range 0–0.243 mg/l). The serum folate, lactate and vitamin B12 levels were normal. Antinuclear antibodies and anti-double stranded DNA antibodies were negative. The patient was positive for an antineutrophil cytoplasmic antibody. Vascular ultrasound was normal. Brain Magnetic Resonance Imaging (MRI) showed left hemisphere atrophy, subdural effusion, widespread calcification foci, hemorrhagic foci and local vasculitis (Figure 1A). Due to the possible diagnosis of autoimmune-associated vasculitis, the patient was then transferred to our rheumatic department. No specific genetic mutations were found after whole-genome sequencing was completed. Skin biopsy of papules on the left lower limb and right upper limb revealed dermal necrosis, thickening of the vascular wall, local hyperkeratosis and hypokeratosis, as well as perivascular lymphocytic infiltration in the upper dermis without mucin disposition (Figure 2). The diagnosis of Degos disease was made based on characteristic papules, disease course and histopathological findings. A methylprednisolone pulse (20 mg/kg/d), oral prednisone (2 mg/kg), intravenous immunoglobulin (IVIG) (2 g/kg), intravenous cyclophosphamide (0.8 g/m^2^) and aspirin were prescribed. The skin lesions recovered, but there was no significant improvement in neurological manifestations after 1 month of combined treatment. Repeated brain MRI showed lacunar infarction, and the size of the ischemic area increased (Figure 1B). Second doses of methylprednisolone pulse, IVIG, and intravenous cyclophosphamide were prescribed. Approximately half a month later, involuntary movement of both lower limbs was more frequent, and repeated brain MRI showed an increased amount of subdural effusion (Figure 1C). Later, the patient underwent surgery involving the right blurred hole on the right scalp with external drainage. The specimen collected during surgery showed inflammatory cell infiltration (Figure 3). Oral hydroxychloroquine (5 mg/kg), thalidomide (3 mg/kg), and methotrexate (10 mg/m^2^) were added as prednisolone-sparing agents. However, the neurological symptoms of the patient deteriorated to lower limb paralysis and areflexia. Her parent asked for discharge and was not followed up later. Unfortunately, the patient died 10 months after the first initiation.

## Literature review

A literature search for malignant atrophic papulosis, Degos disease and pediatric was carried out using PubMed database until April 2024. The search yielded 34 cases and the general information was summarized in Table 1 (2–28). According to these studies, 15 were female and 19 were male. Median age of disease onset was 7.52 ± 1.14 years.

**Table 1:** Data of pediatric malignant atrophic paplosis cases from previous studies.

Feature	Number (%) or median [IQR]
Number of patients	34
Male/female ratio	15/18 (1:1.2)
Age at diagnosis	7.52 ± 1.14 years
Family history	2 (5.88%)
Skin lesions	34 (100%)
Systemic manifestation	
Gastrointestinal tract	18 (52.9%)
Central nervous system involvement	22 (64.7%)
Lung	2 (5.88%)
Eye	7 (20.6%)
Heart	2 (5.88%)
Kidnsy	1 (2.94%)
Aneurysms	1 (2.94%)
Mortality	24 (70.6%)

In two patients, disease onset soon after birth. Skin lesions were the most frequent clinical manifestation, with a frequency of 100%. Central nervous system and gastrointestinal tract involvements were common. Other system like eye, heart and kidney could also be affected. The mortality rate of Degos disease was high which is almost 70%.

Even treated with eculizumab, neurological symptoms exacerbated and patient died. Most patients died due to neurological involvement.

## Discussion

Degos disease, also known as malignant atrophic papulosis, typically presents with erythematous papules that evolve into porcelain-white atrophic papules surrounded by erythematous rims. These lesions are asymptomatic and subside. The papules range in size from 0.5 to 1 cm in diameter, with the trunk and extremities being the most commonly affected areas (29). The most commonly affected systems are the gastrointestinal system (73%) and nervous system (20%–64%) (29). In pediatrics, there is a preference for nervous system involvement (30). Other systems, such as the optic, cardiovascular, pulmonary and hepatorenal systems, could also be affected.

The first case of neurological involvement in Degos disease was reported in 1960 by Nomland and Layton (31). The clinical manifestations of Neuro-MAP can be divided into central nervous system (CNS) presentations and peripheral nervous system (PNS) presentations. CNS involvements are more common (14). CNS presentations can be divided into parenchymal forms accompanied by meningoencephalitis and meningomyelitis and neurovascular forms accompanied by hemorrhage, infarction and thrombosis. PNS manifestations may include neuropathy, myopathy, or polyradiculopathy (32). The patient in our study had both CNS (intracranial hemorrhage, infarction and thrombosis) and PNS (facial paralysis) involvement.

Histopathological examination revealed hyperkeratosis, atrophy of the epidermis, dermo-epidermal separation and edema (33). Cerebrospinal fluid analysis revealed pleocytosis with elevated protein levels, while CSF glucose levels were within normal range, and CSF culture yielded negative results. Ischemic foci can be observed via brain MRI, regarded as the gold standard (32). Given the typical papules and severe neurological involvement, along with skin biopsy findings indicative of vasculitis, the patient was diagnosed with Degos disease.

Degos disease poses a therapeutic challenge, lacking uniform treatment options. There are few cases in which combined therapy comprising IVIG, prednisolone, and cyclophosphamide can induce remission; therefore, a high dose of IVIG and steroids followed by cyclophosphamide was initiated in our patient. Patients with Degos disease may present with elevated fibrinogen levels in plasma, aggravated platelet aggregation and reduced fibrinolytic activity, and previous studies have reported that antiplatelet agents may be useful in resolving skin lesions (29); therefore, antiplatelet agents (aspirin) were added to the patient's regimen. Unfortunately, despite the combined therapy, the patient's symptoms progressed, leading to a fatal outcome.

Recent studies have shown that Eculizumab which blocks terminal complement, has effects on skin and gastrointestinal lesions, and treprostinil, which is a prostacyclin analog, is effective for treating gastrointestinal (GI) and central nervous system manifestations (34). More advanced therapeutic therapies are needed.

For patients with neurological involvement, the average time from papulosis occurrence to death is 2 years (ranging from 1 to 12 years) (29). Unfortunately, in the case of the patient in our study, recommended treatment and follow-up were not adhered to, and the exact cause of death remained unclear. The patient succumbed to neurological complications associated with Degos disease.

Our study is unique because Degos disease is rarer in children than in infants. There are few cases of infantile Degos disease reported worldwide, and this is the first case reported in China. Degos disease is rare and urgently diagnosed, and new treatment options, such as eculizumab and treprostinil, are imperative. Our case also highlighted that neurologists should consider autoinflammatory diseases when neurological and dermatological manifestations coexist.

## Conclusions

Degos disease is a rare and challenging condition to diagnose. Rapid and accurate diagnosis is crucial, and there is a pressing need for additional treatment options and novel medications to improve prognosis in affected individuals.

## Data Availability

The datasets presented in this article are not readily available because of ethical and privacy restrictions. Requests to access the datasets should be directed to the corresponding author.
